# Impairment of hippocampal long-term potentiation by soluble amyloid-β oligomers is mediated by glutamate transporter 1 expressed in neurons

**DOI:** 10.4103/NRR.NRR-D-24-00882

**Published:** 2025-03-25

**Authors:** Shaomin Li, Jianlin Wang, Qianqin Guo, Yunxin Bai, Wen Liu, Kevin J. Hodgetts, Paul A. Rosenberg, Dennis J. Selkoe

**Affiliations:** 1 Ann Romney Center for Neurologic Diseases, Department of Neurology, Brigham and Women’s Hospital and Harvard Medical School, Boston, MA, USA; 2 Department of Neurology and the F. M. Kirby Neurobiology Center, Boston Children’s Hospital, Boston, MA, USA; 3 Laboratory for Drug Discovery in Neurodegeneration, Brigham & Women’s Hospital and Harvard Medical School, Cambridge, MA, USA

**Keywords:** Alzheimer’s disease, ceftriaxone, glutamate transporter 1, hippocampal synaptic plasticity, long-term potentiation, mitochondria, presynaptic, soluble Aβ oligomers

## Abstract

In Alzheimer’s disease, perturbations of glutamate neurotransmission lead to synaptic dysfunction and synapse loss. Several studies have used glutamate transport inhibitors to demonstrate that soluble oligomers of amyloid-β induce synaptic dysfunction by interrupting glutamate uptake mediated by glutamate transporter 1, the major glutamate transporter in the brain. The cellular targets of the synaptic effects of soluble amyloid-β oligomers, including the nature of any interaction with glutamate transporter 1, remain ill-defined. We have generated a conditional glutamate transporter 1 knockout mouse to investigate cell-type specific functions of glutamate transporter 1. Field excitatory postsynaptic potentials were examined in the CA1 region of mouse hippocampal slices. We confirmed that hippocampal long-term potentiation impairment is induced by both soluble amyloid-β oligomers and glutamate uptake inhibitors. Amyloid-β oligomers, including those isolated directly from the cortex of patients with Alzheimer’s disease, failed to inhibit hippocampal long-term potentiation in neuronal glutamate transporter 1 but not astrocytic glutamate transporter 1 knockout mice. The masking or occlusion of the effect of soluble amyloid-β oligomers by knockout of glutamate transporter 1 in neurons suggests that the metabolic or signaling consequences of knockout of glutamate transporter 1 in neurons and amyloid-β oligomer inhibition of synaptic plasticity show epistasis and thus share a similar molecular pathway. To extend these observations, we tested the effects of other types of manipulation of glutamate homeostasis on synaptic plasticity and the pathophysiology of soluble amyloid-β oligomers. Ceftriaxone, which upregulates glutamate transporter 1 levels, among other effects, prevented the impairment of long-term potentiation by soluble amyloid-β oligomers. Collectively, our findings suggest that the effects of amyloid-β on synaptic function are highly dependent on glutamate reuptake homeostasis and that the disruption of synaptic function by soluble amyloid-β oligomers is mediated by pathways linked to neuronal, not astrocytic, glutamate transporter 1. The findings of this study highlight the translational potential of targeting neuronal glutamate transporter 1 to counteract amyloid-β–induced synaptic dysfunction in Alzheimer’s disease. By showing that glutamate transporter 1 upregulation (e.g., via ceftriaxone) can prevent amyloid-β-related impairments, this research supports developing therapies aimed at modulating glutamate homeostasis to preserve synaptic function and combat cognitive decline in patients with Alzheimer’s disease.

## Graphical abstract

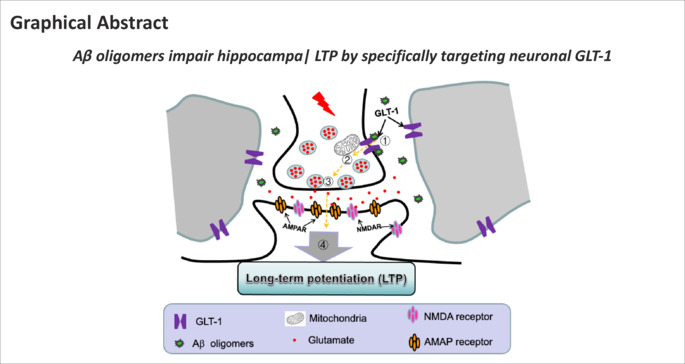

## Introduction

Alzheimer’s disease (AD) is the most common cause of progressive intellectual failure in aged humans and constitutes an enormous public health burden. Effective treatments to change the biological trajectory of AD and slow its clinical progression are just coming into use. The pathological hallmarks of AD are extracellular amyloid plaques formed by aggregation of amyloid-β (Aβ) and intraneuronal neurofibrillary tangles formed by phosphorylated tau proteins. Neuropathological and biomarker studies indicate that a rise in Aβ occurs well prior to and helps drive the accumulation of phosphorylated tau (Buchhave et al., 2012). Aβ-induced aberrant hyperexcitability has been reported in cortical and hippocampal neuronal networks of patients and mouse models of AD (Busche et al., 2012; Palop and Mucke, 2016; Samudra et al., 2023). Memantine, which is an FDA-approved drug for AD that reduces excess glutamatergic activity, showed some symptomatic benefits for some AD patients but did not produce much improvement in cognition (Li et al., 2019). Targeting the alteration in glutamate homeostasis that occurs in AD, instead of blocking excessive glutamatergic activity, may be more effective in ameliorating the prodromal stage of AD.

Glutamate homeostasis plays an essential role in learning and memory by regulating synaptic plasticity and neural circuit function (Valtcheva and Venance, 2019; Collingridge and Abraham, 2022; Chakraborty et al., 2023). Excessive and prolonged excitatory glutamatergic activity could lead to excitotoxic neuronal death (Karki et al., 2018; Choi, 2020), which has been proposed to be important in the pathogenesis of neurodegenerative diseases such as AD (Armada-Moreira et al., 2020). Among the glutamate transporters, the excitatory amino acid transporter 2 (EAAT2) also called glutamate transporter 1 (GLT-1) accounts for 90%–95% of glutamate uptake in synaptosome preparations from rodent forebrain. It regulates glutamatergic signaling and provides for the reuptake of synaptically released glutamate by both adjacent astrocytes and axon terminals (McNair et al., 2019, 2020; Pajarillo et al., 2019; Rimmele et al., 2021). Most GLT-1 is expressed in astrocytes, while 5%–10% of GLT-1 is expressed in neurons, primarily in axon terminals (Petr et al., 2015; Rimmele and Rosenberg, 2016). Although the neuronal GLT-1 (nGLT-1) is only a small portion of the total (Furness et al., 2008; Petr et al., 2015), it makes a major contribution to synaptosomal glutamate uptake (Petr et al., 2015; Rimmele and Rosenberg, 2016; Zhou et al., 2019), even in crude synaptosomal preparations (Petr et al., 2015), and appears to have an important metabolic role in providing synaptic mitochondria with glutamate, which is an important substrate (McNair et al., 2019, 2020). To date, most studies regarding GLT-1 focus on astrocytic GLT-1 (aGLT-1), whereas the function of nGLT-1 has been little explored.

We previously reported that soluble, diffusible Aβ oligomers (oAβ) potently impaired hippocampal synaptic plasticity by interfering with glutamate reuptake (Lei et al., 2016; Li et al., 2009, 2011). These findings have been confirmed and extended by several other groups (Dutar and Potier, 2019; Zott et al., 2019). Furthermore, increasing evidence has shown that GLT-1 functional loss is common in patients with early AD (Jacob et al., 2007; Scott et al., 2011) and in an animal model of AD (Meeker et al., 2015). Consistent with these findings, upregulating GLT-1 expression by ceftriaxone and similar “LDN” compounds has been shown to improve or protect against AD-type cognitive impairment or delay the onset of AD-like symptoms in animal models (Takahashi et al., 2015; Foster et al., 2019). However, the detailed mechanism by which Aβ oligomers alter the function of glutamate transporters remains unknown.

Although the main function of GLT-1 is likely to be clearance of glutamate from the extracellular space, there may be additional functions of GLT-1 that have yet to be fully appreciated. In particular, since GLT-1 expressed in axon terminals represents only 5%–10% of the total, it is not clear whether GLT-1 in axon terminals makes a significant contribution to glutamate clearance or whether it has additional functions in this location. To pursue the question of cell-type specific functions of GLT-1, we generated a conditional GLT-1 knockout mouse, and lines lacking GLT-1 expression in either astrocytes (aKO) or neurons (nKO) were produced using Cre/lox technology. Here we characterize aGLT-1 and nGLT-1 knockout mice to determine the role of each isoform on the effects of oligomeric Aβ on hippocampal synaptic plasticity.

## Methods

### Mice

Both male and female conditional GLT-1 knockout mice were obtained from the founding colony at Boston Children’s Hospital (Slc1A2^tm1.1Pros^; MGI: 5752263) (Petr et al., 2015). Neuronal GLT-1 knockout mice were generated in which the *GLT-1* gene was inactivated in neurons by expression of synapsin-Cre as described previously (*GLT-1*^flox/flox^; synapsin-Cre) (Petr et al., 2015; Fischer et al., 2018), and littermate controls with normal GLT-1 function (GLT-1^flox/flox^). These are referred to in the current paper as synGLT-1 KO (neuronal GLT-1 KO) and wild-type littermate controls, respectively. To determine that our observations were not due to Cre-recombinase expression, *per se* (Honore et al., 1988), we generated and used synapsin-Cre control mice. Male mice with a tamoxifen-inducible astrocyte-specific knockout of GLT-1 (GLT-1^Δ/Δ^;GFAP-CreER^T2^) are referred to as gfapGLT-1 KO and were generated using the hGFAP-CreER^T2^ driver (Casper et al., 2007) as described previously (Petr et al., 2015). Pups were treated daily with tamoxifen (T5648, Sigma, St. Louis, MO, USA; 33 mg/kg, intraperitoneal (i.p.) or oral gavage in sunflower oil) starting from P5 and for 4–5 consecutive days (Ganat et al., 2006). Tamoxifen solutions were made fresh for a given litter and never frozen. Experiments were conducted on adult male mice, neuronal GLT-1 KO experiments were performed with three age groups, 6–40 weeks, using age-matched littermates as controls; gfapGLT-1 KO experiments were performed with 6–40 week old male mice and tamoxifen-treated littermate controls. Other WT mice (male and female) contained a mixed background of C57BL/6 and 129. Animals were housed in a temperature-controlled room on a 12-hour light/dark cycle and had ad libitum access to food and water. All animal experiments were performed in accordance with NIH guidelines, and were approved by the Children’s Hospital Boston (00001645, April 8, 2022) and Brigham Women’s Hospital (2016N000342, March 21, 2018) Institutional Animal Care and Use Committee.

### Hippocampal slice preparation

Mice were euthanized with isoflurane (2% with oxygen inhalation, Henry Schein Animal Health, Dublin, OH, USA) at 2 to 8 months of age. Brains were quickly removed and submerged in ice-cold oxygenated sucrose-replaced artificial cerebrospinal fluid (ACSF) cutting solution (in mM) (206 sucrose, 2 KCl, 2 MgSO_4_, 1.25 NaH_2_PO_4_, 1 CaCl_2_, 1 MgCl_2_, 26 NaHCO_3_, 10 D-glucose, pH 7.4, 315 mOsm, all compounds were purchased from Sigma (St. Louis, MO, USA)). Transverse slices (350 μm thickness) from the middle portion of each hippocampus were cut with a vibroslicer (A Biomedical Service, Fresno, CA, USA). After dissection, slices were incubated in ACSF that contained the following (in mM): 124 NaCl, 2 KCl, 2 MgSO_4_, 1.25 NaH_2_PO_4_, 2.5 CaCl_2_, 26 NaHCO_3_, 10 D-glucose, pH 7.4, 310 mOsm, in which they were allowed to recover for at least 90 minutes before recording. To mitigate glutamate-induced excitotoxicity during the cutting and recovery period in middle-aged neuronal GLT-1 knockout mice, we adjusted the cutting solution as per the method described by Camp et al. (2023) and based on previous work by Ting et al. (2014) with the recipe provided (in mM): 88 sucrose, 80 NaCl, 2.5 KCl, 1.25 Na_2_HPO_4_, 26 NaHCO_3_, 10 D-glucose, 2 thiourea, 3 sodium pyruvate, 5 sodium ascorbate, 12 N-acetylcysteine, 10 MgSO_4_, and 0.5 CaCl_2_. Slices were incubated in the same sucrose-based ACSF as described above but with 4 mM MgSO_4_ at 32°C for 30 minutes then returned to room temperature for at least an hour before use. A single slice was then transferred to the recording chamber and submerged beneath continuously perfusing ACSF that had been saturated with 95% O_2_ and 5% CO_2_. Slices were incubated in the recording chamber for 20 minutes before stimulation under room temperature (**~**26°C).

### Electrophysiological recordings

We used standard procedures to record field excitatory postsynaptic potentials (fEPSP) in the CA1 region of the hippocampus. A bipolar stimulating electrode (FHC Inc., Bowdoin, ME, USA) was placed in the Schaffer collaterals to deliver test and conditioning stimuli. A borosilicate glass recording electrode filled with ACSF was positioned in the stratum radiatum of CA1, 200–300 μm from the stimulating electrode. fEPSP in the CA1 region were induced by test stimuli at 0.05 Hz with an intensity that elicited a fEPSP amplitude 40%–50% of maximum. Test responses were recorded for 30–60 minutes prior to beginning the experiment to assure stability of the response. Once a stable test response was attained, experimental treatments (Aβ species and/or agents) were added to the 10 mL ACSF perfusate, and a baseline was recorded for an additional 30 minutes. To induce long-term potentiation (LTP), two consecutive trains (1 second) of stimuli at 100 Hz separated by 20 seconds were applied to the slices, a protocol that induced LTP lasting approximately 1.5 hours in wild-type mice of this genetic background. The field potentials were amplified 100× using an Axon Instruments 200B amplifier (Axon Instruments, San Jose, CA, USA) and digitized with Digidata 1322A (Axon Instruments). Data were sampled at 10 kHz and filtered at 2 kHz. Traces were obtained by pClamp 9.2 (Axon Instruments, San Jose, CA, USA) and analyzed using the Clampfit 9.2 program (Axon Instruments, San Jose, CA, USA). LTP values reported throughout were measured at 60 minutes after the conditioning stimulus unless stated otherwise. Paired pulse facilitation of fEPSP was tested by two stimuli 50 ms apart.

### Human brain homogenate preparation

Homogenates of human brains (**Additional Table 1**) were prepared as described elsewhere (Shankar et al., 2008; Yang et al., 2017). Human brains were obtained from a brain bank. Individual consent forms were not required. Frozen brain tissue collected at Massachusetts Alzheimer’s Disease Research Center (MADRC Neuropathology Core, Harvard, MA, USA) and Brigham and Women’s Hospital under the Partners Institutional Review Board of the Partners Human Research Committee-approved protocols (2020P002649, September 3, 2020). Frozen samples of temporal or frontal cortex (1 g) were allowed to thaw on ice, chopped into small pieces with a razor blade, and then homogenized with 25 strokes of a Dounce homogenizer (Fisher, Ottawa, ON, Canada) in 4 mL ice-cold 20 mM Tris–HCl, pH 7.4, containing 150 mM NaCl (Tris-buffered saline [TBS]) and protease inhibitors. Water-soluble Aβ was separated from membrane-bound and plaque Aβ by centrifugation at 175,000 × *g* and 4°C in a TLA 100 rotor (Beckman Coulter, Fullerton, CA, USA) for 30 minutes, and the supernatant (referred to as TBS extract) aliquoted and stored at –80°C.

**Additional Table 1:** Demographic details of cases in this study

Case	Age	Sex	Clinical diagnosis	Neuropathological diagnosis
**AD-1**	83	Female	AD	AD with CAA
**AD-2**	84	Female	AD	AD with Limbic LBD, CAA
**AD-3**	67	Male	AD	AD with diffuse neocortical LBD
**Control1**	69	Male	Dementia	Multi-infarct dementia
**Control2**	64	Male	CJD	Cerebellar degeneration

AD: Alzheimer’s disease; CAA: cerebral amyloid angiopathy; LBD: Lewy body dementia; CJD: Creutzfeldt-Jakob disease.

### Cellular amyloid-β (7PA2 CM) preparations

Cellular secreted Aβ peptides were collected and prepared from the conditioned medium (CM) of a CHO cell line (7PA2) that stably expresses human APP751 containing the V717F AD mutation (Podlisny et al., 1995). Cells were grown in Dulbecco’s modified Eagle’s medium (DMEM) containing 10% fetal bovine serum, 1% penicillin/streptomycin, 2 mM L-glutamine, and 200 mg/ml G418 for selection. Upon reaching **~**95% confluency, the cells were washed and cultured overnight (15 hours) in serum-free medium. CM was collected, spun at 1500 × *g* to remove dead cells and debris, and stored at 4°C. The CM was concentrated 10-fold with a YM-3 Centricon filter (Millipore Sigma, Burlington, MA, USA). Aliquots of concentrated 7PA2 CM were stored at –80°C.

### Drug administration

Paired control and experimental hippocampal slices from a single mouse were maintained together in a single chamber, except during individual drug treatments. The following compounds were purchased from Tocris (Minneapolis, MN, USA): DL-threo-β-benzyloxyaspartic acid (TBOA), WAY 213613, LDN 212320 and LDN 0215111 and EUK-134, all of which were dissolved in dimethylsulfoxide (DMSO). Dihydrokainic acid (DHK) was dissolved in ACSF. Ceftriaxone (CEF) was purchased from Sigma (St. Louis, MO, USA). AβS26C-dimer was purchased from JPT peptide technologies (JPT, Berlin, Germany). The DMSO concentration used in experiments was < 0.01%. All compounds were added to the perfusion ACSF for at least a 20-minute baseline recording before high-frequency stimulation (HFS) was applied. When combined with oAβ (AD TBS extract, 7PA2 CM, or AβS26C-dimer), these compounds were added 10 minutes or 2 hours before the administration of oAβ and then the baseline was recorded for at least 30 minutes prior to HFS. The compounds and oAβ were then perfused for the entire duration of the recording.

### Statistical analysis

GraphPad Prism (Version 9.0.2; GraphPad, San Diego, CA, USA) was used for data visualization and statistical analyses. Unless otherwise specified, LTP values represent measurements taken 60 minutes after the conditioning stimulus. Results are expressed as means ± SEM from at least four independent biological samples. Statistical significance was assessed using a two-tailed Student’s *t*-test for single comparisons and one-way analysis of variance (ANOVA) for multiple groups. For multiple-group comparisons, one-way or two-way ANOVA with Bonferroni post hoc tests were applied.

## Results

### Similarity of the effect of the glutamate transport inhibitor threo-β-benzyloxyaspartic acid and soluble amyloid-β oligomers in blocking hippocampal long-term potentiation

**Figure 1:**
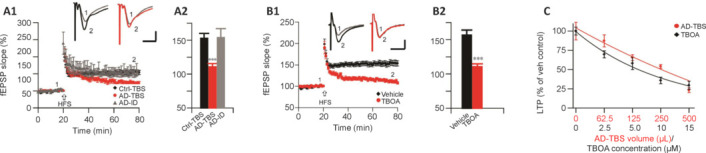
Hippocampal LTP impairment induced by glutamate uptake inhibitors and soluble Aβ oligomers may share similar pathways. (A1, A2) Hippocampal LTP was inhibited by AD brain TBS extract (*n* = 9), not by control brain TBS extract (*n* = 10) or AD-ID (*n* = 7); A2 is summary data from A1 (one-way analysis of variance with Bonferroni *post hoc* tests). (B1, B2) LTP was inhibited by DL-TBOA (10 µM, *n* = 10 *vs*. Veh: *n* = 10, two-tailed Student’s *t*-test); B2 is summary data from B1. (C) Dose-dependent LTP inhibition by AD TBS extract and DL-TBOA. These data are consistent with the hypothesis that both Aβ oligomers and TBOA may target the same pathway. Inset traces (A1 and B1) show typical fEPSPs recorded before (gray, labeled as 1) and after (black/red, labeled as 2) high-frequency stimulation stimulus for each condition. Horizontal calibration bar 10 ms; vertical bar 0.5 mV. ***P < 0.001, *vs*. control or vehicle group. AD: Alzheimer’s disease; Ctrl: control; fEPSP: field excitatory postsynaptic potentials; ID: immunodepleted; LTP: long-term potentiation; TBOA: DL-threo-β-benzyloxyaspartic acid; TBS: Tris-buffered saline.

We and others have obtained evidence that diffusible oAβ impairs synaptic plasticity by inhibiting glutamate transport function (Li et al., 2009; Zott et al., 2019). Here, we first repeated our previous work showing that soluble oAβ from AD brain aqueous (TBS) extracts impair hippocampal LTP, whereas both control brain TBS extracts and AD brain extracts immunodepleted 3 times sequentially of Aβ (AD-ID) had no effect on (**Figure 1A1** and **A2**). TBOA at 10 µM, a concentration that would be expected to partially inhibit GLT-1 and excitatory amino acid carrier 1, but not significantly inhibit glutamate aspartate transporter, also impaired hippocampal LTP (**Figure 1B1** and **B2**). Aβ oligomers and TBOA similarly impaired hippocampal LTP in a dose-dependent manner (**Figure 1C**). The maximum concentration we used here is 0.5 mL of AD brain TBS extract (AD TBS) and 20 µM of DL-TBOA. These data show that inhibition of glutamate transport produces similar effects to Aβ on hippocampal LTP.

### Amyloid-β impairment of hippocampal long-term potentiation is not affected by astrocytic glutamate transporter 1 inhibitors

**Figure 2:**
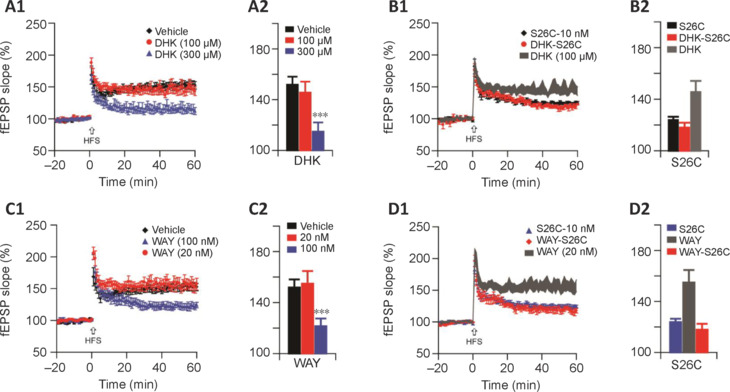
Specific GLT-1 inhibitors fail to prevent soluble Aβ oligomers from impairing hippocampal LTP. (A1, A2) Dose-dependent hippocampal LTP inhibition by the specific GLT-1 inhibitor, DHK (100 µM, *n* = 6 and 300 µM, *n* = 7); A2 is summary data from A1 (one-way analysis of variance with Bonferroni *post hoc* tests). (B1, B2) Low dose DHK (100 µM) has no effect on AβS26C-dimer (10 nM) impairment of LTP (S26C alone, *n* = 6, DHK + S26C, *n* = 7); B2 is summary data from B1 (one-way ANOVA with Bonferroni *post hoc* tests). (C1, C2) Dose-dependent hippocampal LTP inhibition by GLT-1 selective inhibitor, WAY 213613 (20 nM, *n* = 8 *vs*. 100 nM, *n* = 6); C2 is summary data from C1 (one-way ANOVA with Bonferroni *post hoc* tests). (D1, D2) A low dose WAY 213613 (20 nM) did not prevent AβS26C-dimer induced impairment of LTP (*n* = 8); D2 is summary data from D1 (one-way ANOVA with Bonferroni *post hoc* tests). ***P < 0.001, *vs*. control or vehicle group. Aβ: Amyloid-β; fEPSP: field excitatory postsynaptic potentials; GLT-1: glutamate transporter 1; LTP: long-term potentiation.

As EAAT2 (GLT-1) is the most abundant glutamate transporter in the brain and plays a key role in clearing glutamate following its release into the extracellular space, we chose well-characterized GLT-1 selective inhibitors, DHK (Ki 23 µM for EAAT2 and > 3 mM for EAAT1, EAAT3) and WAY-213613 (IC50 values are 85, 3787 and 5004 nM for EAAT2, EAAT3 and EAAT1, respectively) (Dunlop et al., 2005; Greenfield et al., 2005) to study the role of GLT-1 in Aβ-mediated synaptic dysfunction (**Figure 2**). DHK inhibits GLT-1 expressed in both astrocytes and neurons (Danbolt, 2001; Rimmele and Rosenberg, 2016). A recent study showed that a relatively low dose (50–100 µM) of DHK did not alter hippocampal LTP (Barnes et al., 2020). We found that 100 µM of DHK had no effect on LTP but 300 µM blocked it (**Figure 2A1** and **A2**). We tested whether low-dose DHK affected the impairment of LTP by oAβ. Synthetic AβS26C crosslinked dimers still impair LTP even in the presence of 100 µM DHK in wild-type animals (**Figure 2B1** and **B2**).

We next tested another GLT-1 selective blocker, WAY-213613 (Dunlop et al., 2005). This compound also showed a dose-dependent LTP inhibition (**Figure 2C1** and **C2**). As with DHK, a low dose (20 nM) of WAY-213613 did not prevent the impairment of LTP by AβS26C-dimers (**Figure 2D1** and **D2**). To further confirm the effects of synthetic soluble Aβ oligomers on WAY-213613 mediated LTP, we tested another well-studied source of cell-secreted soluble oligomers of Aβ. These oligomers are present in the conditioned media (CM) of CHO cells stably expressing FAD-mutant (V717F) human APP (i.e., 7PA2 cells) (Li et al., 2009, 2011; Lei et al., 2016). This source of Aβ oligomers impaired LTP, which was not rescued by a low dose of WAY213613 (**Additional Figure 1**). Taken together, the results obtained with DHK and WAY213613 suggest that partial pharmacological inhibition of glutamate clearance, a function subsumed predominantly by astrocytic GLT-1, does not mimic the effects of oAβ on impairment of hippocampal LTP.

### Impairment of hippocampal long-term potentiation by soluble amyloid-β oligomers may result from an effect specifically on neuronal glutamate transporter 1, not astrocytic glutamate transporter 1

**Figure 3:**
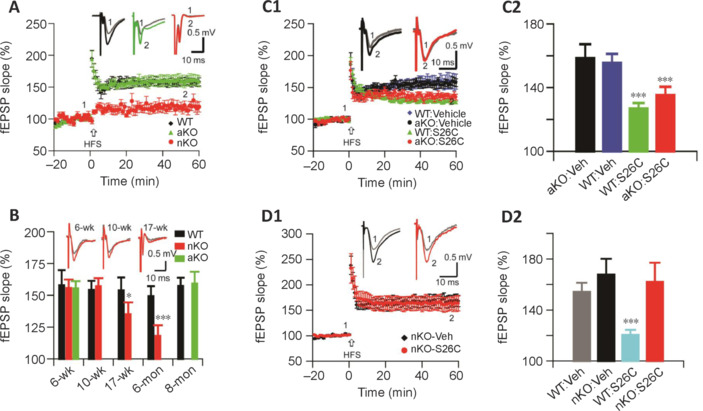
Hippocampal LTP in nKO mice is resistant to the effects of soluble Aβ oligomers. (A) Hippocampal LTP was recorded in slices from 8-month-old aKO mice (green, *n* = 14) and 6-month-old nKO mice (red, *n* = 9) are compared to 8-month-old WT mice (black, *n* = 9). (B) Summary data of hippocampal LTP recorded from aKO and nKO mice in different ages. LTP was normal at 10-week nKO mice (*n* = 9), but was partially impaired in 17-week-old mice (*n* = 12), and severely impaired in 6-month mice (*n* = 9) (one-way ANOVA with Bonferroni *post hoc* tests). (C1, C2) Slices from young (6–10 weeks old) aKO mice showed normal hippocampal LTP (black, *n* = 9), and this LTP was sensitive to S26C (red, *n* = 8) as also in WT slices (green, *n* = 9); C2 is summary data from C1 (one-way analysis of variance with Bonferroni *post hoc* tests). (D1, D2) Slices from 10-week-old nKO mice have normal LTP (black, *n* = 6) that was not inhibited by AβS26C dimer (10 nM, red, *n* = 6). D2 is summary data from D1 (one-way analysis of variance with Bonferroni *post hoc* tests). Inset traces (A, C1, and D1) represent typical fEPSPs recorded before (gray, labeled as 1) and after (black/green/red, labeled as 2) high-frequency stimulation stimulus for each condition. Horizontal calibration bar 10 ms; vertical bar 0.5 mV. *P < 0.05, ***P < 0.001, *vs*. control or vehicle group. Aβ: Amyloid-β; fEPSP: field excitatory postsynaptic potentials; GLT-1: glutamate transporter 1; LTP: long-term potentiation; nKO: neuronal GLT-1-KO; WT: wild-type.

Previously it was thought that GLT-1 is expressed exclusively in astrocytes, but we and others showed that GLT-1 is also present in axon terminals (Furness et al., 2008; Petr et al., 2015). We were interested in the question of whether astrocytic GLT-1 or neuronal GLT-1 is implicated in the regulation of synaptic plasticity. We used two lines of conditional GLT-1-KO mice – astrocytic GLT-1-KO (aKO) and neuronal GLT-1-KO (nKO) – produced using tamoxifen-inducible GFAP-CreERT2 or synapsin-Cre, respectively (Petr et al., 2015). We found that hippocampal LTP was not altered in aKO mice from 6 weeks old to 8 months old. In contrast, hippocampal LTP was impaired in nKO mice in an age-dependent manner (**Figure 3A** and **B**). LTP was normal at 10-week nKO mice, but was partially impaired in 17-week mice, and severely impaired in 6-month mice. Over this period of time, it was found that recovery of function in slices from the neuronal GLT-1 knockout was impaired, resulting in small or undetectable fEPSPs (see inset, **Figure 3B**). The impaired recovery was found to be due to excitotoxic injury (Rimmele et al., 2021). It is possible that LTP deficits in slices from adult nKO mice are due to the synaptic injury caused by increased vulnerability to glutamate excitotoxicity (Rimmele et al., 2021).

When we added the soluble oAβ - AβS26C dimers (10 nM) to brain slices from 6–9 week old aKO, LTP was impaired as in their WT littermates (**Figure 3C1** and **C2**). Another source of Aβ oligomers, enriched 7PA2 CM, also impaired LTP in aGLT-1 KO mice as in their WT littermates (**Additional Figure 2A** and **B**).

Young (10 week) nGLT-1 KO mice had normal hippocampal LTP (**Figure 3B**). Interestingly, Aβ oligomers (S26C dimers) failed to inhibit hippocampal LTP in 10-week nGLT-1 KO mice (**Figure 3D1** and **D2**). To further study the age dependence of the Aβ effect in the nGLT-1 KO mice, we tested younger (6 week old) nGLT-1 KO mice. These younger nKO mice showed normal LTP. AβS26C dimers (10 nM) inhibited hippocampal LTP in brain slices from age-matched WT mice, but the same dose of Aβ S26C-dimers failed to inhibit LTP in the slices from nKO mice (WT: 121.3% ± 3.1%, *n* = 7 *vs.* nKO: 139.3% ± 2.7%, *n* = 6, *P* < 0.01; **Additional Figure 2C**). Similarly, oAβ-rich AD brain aqueous extracts impaired hippocampal LTP in WT mice but failed to inhibit LTP in 6-week nKO littermate mice (WT: 125.0% ± 3.8%, *n* = 7 *vs*. nKO: 140.8.3% ± 6.9%, *n* = 7, *P* < 0.05; **Additional Figure 2C**). These data suggest that the impairment of LTP by soluble Aβ oligomers specifically involves a pathway that is perturbed in the neuronal GLT-1 knockout.

### Impairment of hippocampal long-term potentiation by soluble amyloid-β oligomers is prevented in neuronal glutamate transporter 1 knockout slices treated with EUK-134

To ensure that the altered response to soluble oAβ neuronal GLT-1 knockout (nGLT-1 KO) slices was not due to excitotoxic injury (Rimmele et al., 2021), we used modified cutting and recovery buffers optimized for adult and aging animals, following the method by Camp et al. (2023) and based on previous work by Ting et al. (2014). We also tested the effects of supplementing the recovery solution with either MK-801, a non-competitive, slowly reversible NMDA receptor channel blocker (10 µM), or EUK-134 (30 µM), a superoxide dismutase mimic known to protect nGLT-1 KO slices from excitotoxic injury and promote recovery (Rong et al., 1999; Li et al., 2024).

**Figure 4:**
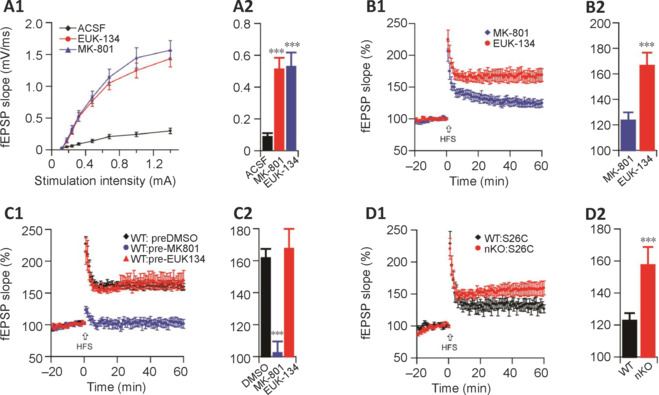
Synaptic function recovered brain slices of nGLT-1 KO mice are resistant to the effects of soluble Aβ oligomers. (A1, A2) The impaired excitatory synaptic transmission in adult nGLT-1 KO mice was successfully restored by incubation with either MK-801 (10 µM, *n* = 12, shown in blue) or EUK-134 (30 µM, *n* = 19, shown in red); A2 is summary data from A1 at a stimulation intensity of 0.48 mA (one-way analysis of variance with Bonferroni *post hoc* tests). (B1, B2) Hippocampal LTP induced by high-frequency stimulation occurred in slices recovered by EUK-134 (red, *n* = 10) but not in those recovered by MK-801 (blue, *n* = 10) in nGLT-1 KO mice; B2 is summary data from B1 (two-tailed Student’s *t*-test). (C1, C2) LTP in the hippocampus, induced by high-frequency stimulation, was observed *in slices* pre-treated with EUK-134 (red, *n* = 7), similar to the control treated with the vehicle alone (black, *n* = 10), but not in those treated with MK-801 (blue, *n* = 7) in wild-type mice; C2 is summary data from C1 (one-way ANOVA with Bonferroni *post hoc* tests). (D1, D2) Recordings of hippocampal LTP in slices recovered with EUK-134 (30 µM) exposed to AβS26C-dimer (10 nM) showed results in nGLT-1 KO (red, *n* = 8) but not in WT (black, *n* = 5) mice. D2 is summary data from D1 (two-tailed Student’s *t*-test). ***P < 0.001, *vs*. control or vehicle group. ACSF: Artificial cerebrospinal fluid; Aβ: amyloid-β; fEPSP: field excitatory postsynaptic potentials; GLT-1: glutamate transporter 1; LTP: long-term potentiation; nGLT-1: neuronal GLT-1; nKO: neuronal GLT-1-KO; WT: wild-type.

Both MK-801 and EUK-134 restored impaired fEPSPs during the cutting-induced injury as illustrated in **Figure 4A**. However, in the recovered nGLT-1 KO brain slices, typical hippocampal LTP was successfully elicited only in those treated with EUK-134, but not with MK-801, under standard ACSF conditions (**Figure 4B1** and **B2**). We concluded that MK-801 failed to allow LTP recovery due to its residual blockade of NMDA receptors during recording, a known effect of its slow unblocking kinetics (Kloog et al., 1988). Further evidence supporting this hypothesis comes from wild-type mouse slices pretreated with MK801 (10 µM), which showed complete LTP blockade (**Figure 4C1** and **C2**). In contrast, EUK-134 (30 µM) pretreatment did not affect LTP, similar to the control treated with the vehicle alone (**Figure 4C1** and **C2**). The partial LTP observed in nGLT-1 KO mice pre-treated with MK801 (**Figure 4B1** and **B2**) compared to wild-type mice (**Figure 4C**) may indicate elevated extracellular glutamate levels in nGLT-1 KO mice.

Exposure to synthetic AβS26C-dimer (**Figure 4D1** and **D2**) or AD-TBS (**Additional Figure 3**) in the recording buffer caused impairment of LTP generation in slices from WT animals but not in slices from the nGLT-1 KO (treated with EUK-134 to promote recovery), providing additional evidence to support the hypothesis that soluble Aβ oligomers disrupt hippocampal LTP in brain slices from wild-type mice while having no discernible effect on slices from the nGLT-1 mice, even under conditions promoting full functional recovery of those slices. These findings provide additional evidence that soluble Aβ oligomers interfere with hippocampal LTP through a mechanism related to the function of GLT-1 expressed in axon terminals.

### Ceftriaxone, a glutamate transporter up-regulator, restored soluble amyloid-β oligomer impairment of hippocampal long-term potentiation

CEF, an antibiotic that increases glutamate transporter levels, can reverse the damaging effects of soluble oAβ on hippocampal LTP. CEF, which boosts GLT-1 expression via the nuclear factor-κB pathway (Rao et al., 2023), has shown protective effects in various neurodegenerative disease models, including Parkinson’s disease, Alzheimer’s disease, amyotrophic lateral sclerosis, and Huntington’s disease (Yimer et al., 2019).

**Figure 5:**
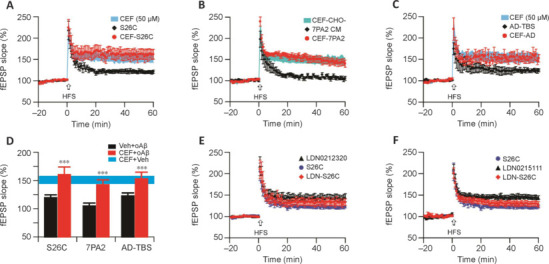
Ceftriaxone prevents impairment of LTP by soluble Aβ oligomers. (A) Ceftriaxone fully restored AβS26C-dimer impaired hippocampal LTP (red, *n* = 5 *vs*. S26C, *n* = 5, *P* < 0.001). (B) Ceftriaxone fully restored 7PA2 CM impaired hippocampal LTP (red, *n* = 6, *vs*. 7PA2 CM, *n* = 5, *P* < 0.001). (C) Ceftriaxone fully restored AD brain extract impaired hippocampal LTP (red, *n* = 6, *vs*. AD TBS, *n*=5, *P* < 0.001). (D) Summary results of ceftriaxone preventing soluble Aβ oligomers impaired LTP (one-way analysis of variance with Bonferroni post *hoc* tests). Pretreatment of LDN/OSU-0212320 (0.5 µM, LDN/OSU-0212320: *n* = 9 *vs.* LDN + S26C, *n* = 8, one-way analysis of variance with Bonferroni *post hoc* tests, *P* < 0.001) (E) and LDN/OSU-0215111 (100 µM, LDN/OSU-0215111, *n* = 7 *vs*. LDN + S26C, *n* = 7, *P* < 0.001) (F) for 4 hours failed to prevent AβS26C-dimer impaired hippocampal LTP (red) (one-way analysis of variance with Bonferroni *post hoc* tests). ***P < 0.001. AD: Alzheimer’s disease; Aβ: amyloid-β; CEF: ceftriaxone; fEPSP: field excitatory postsynaptic potentials; LTP: long-term potentiation; oAβ: soluble Aβ oligomer; TBS: Tris-buffered saline.

To see if CEF can protect against soluble Aβ oligomer damage, we pretreated brain slices with CEF for 2–4 hours and then exposed them to different sources of soluble oAβ. We found that CEF prevented LTP impairment caused by all tested Aβ oligomers: AβS26C dimer (**Figure 5A**), 7PA2 cell-conditioned media (**Figure 5B**), and AD brain extract (**Figure 5C**). Results were summarized and shown in **Figure 5D**.

Additionally, we tested whether upregulating GLT-1 on impairment of hippocampal LTP by oAβ, using two LDN compounds, LDN/OSU-0212320 (Takahashi et al., 2015) (0.5 µM) and LDN/OSU-0215111 (Foster et al., 2019) (100 µM), could prevent oAβ-induced LTP impairment. However, neither compound prevented the damage caused by oAβ, disputed increasing GLT-1 levels (**Figure 5E** and **F**).

### Ceftriaxone prevents soluble amyloid-β oligomer impairment of long-term potentiation through a presynaptic mechanism

**Figure 6:**
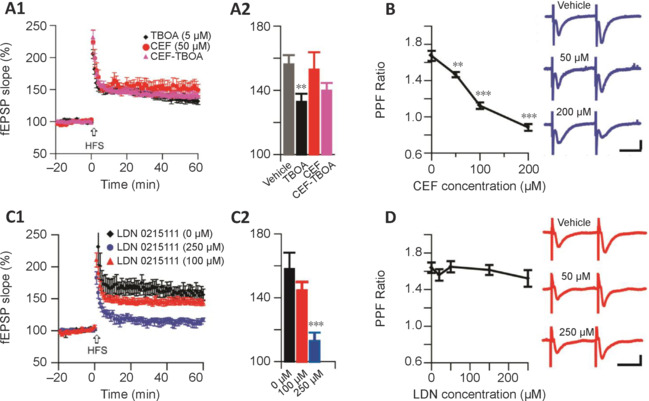
Ceftriaxone interrupts the presynaptic function in acute brain slices. (A1, A2) Ceftriaxone failed to restore LTP impaired by TBOA (5 µM) (pink, *n* = 6, *vs.* TBOA *n* = 6, *P* > 0.05). A2 is summary data from A1 (one-way analysis of variance with Bonferroni *post hoc* tests). (B) Ceftriaxone causes PPF to decrease in a dose-dependent manner (one-way analysis of variance with Bonferroni *post hoc* tests). The right panels were the representative traces. (C) Higher concentration (250 µM) of LDN-0215111 impaired hippocampal LTP (blue, *n* = 5), whereas the lower dose (100 µM) (red, *n* = 7, *vs*. Veh: *n* = 9, *P* > 0.05) exhibited a milder impact. C2 is summary data from C1 (one-way analysis of variance with Bonferroni *post hoc* tests). (D) LDN-0215111 did not alter paired-pulse facilitation decreased in acute brain slices. The right panels were the representative traces. **P < 0.01, ***P < 0.001, *vs*. control or vehicle group. CEF: Ceftriaxone; fEPSP: field excitatory postsynaptic potentials; HFS: high-frequency stimulation; LTP: long-term potentiation; PPF: paired-pulse facilitation; TBOA: DL-threo-β-benzyloxyaspartic acid.

A recent report found that CEF does not speed up glutamate clearance but instead reduces the release of glutamate from presynaptic neurons (Wilkie et al., 2021). To test this further, we pretreated brain slices with CEF for 2 hours, and then added TBOA (5 µM), a glutamate uptake blocker, to see its effect on LTP. We observed no significant difference between the TBOA only group and the CEF + TBOA group, indicating CEF didn’t affect TBOA’s impact on LTP (**Figure 6A1** and **A2**).

Next, we explore how CEF might protect against Aβ-induced LTP impairment by measuring paired-pulse facilitation (PPF), which reflects presynaptic glutamate release. We found that CEF significantly reduced PPF in a dose-dependent manner (Veh: 1.67 ± 0.19, *n* = 13; 50 µM: 1.46 ± 0.11, *n* = 12; 100 µM: 1.12 ± 0.12, *n* = 11 and 200 µM: 0.88 ± 0.14, *n* = 14, **Figure 6B**). In contrast, the LDN-0215111 compound, which inhibits LTP in a dose-dependent manner (**Figure 6C1** and **C2**), did not alter PPF (**Figure 6D**), showing no effect on presynaptic release. These results suggest that CEF may protect against Aβ-induced LTP impairment through a presynaptic mechanism. This aligns with the idea that neuronal GLT-1 (presynaptic-axon dominated), rather than astrocytic GLT-1, plays a role in Aβ-mediated LTP impairment.

## Discussion

The present study demonstrates that impairment of hippocampal LTP by soluble Aβ oligomers is similar to that produced by glutamate transporter inhibitors (TBOA, DHK, and WAY213613). Mechanistically, the Aβ oligomer disruption of synaptic function may be mediated or modulated by neuronal GLT-1, not astrocytic GLT-1, because the Aβ impairment of LTP is not affected by astrocytic GLT-1 KO, but appears to be suppressed by neuronal GLT-1 knockout. Treating with FDA-approved beta-lactam antibiotics (ceftriaxone) can prevent soluble Aβ oligomers impaired LTP via reducing presynaptic release.

Many reports demonstrate that GLT-1 dysfunction or decreased GLT-1 expression is present in AD patients, AD model mice, or directly produced by oAβ (Li et al., 2011; Tong et al., 2017; Brymer et al., 2023). However, most studies assume aGLT-1 is the primary target. Few studies recognize that GLT-1 is also located at presynaptic terminals in neurons (nGLT-1). The possibility of different functions of nGLT-1 and aGLT-1 has only begun to be explored (Petr et al., 2015), but recent reports suggest that nGLT-1 may have a predominantly metabolic function (McNair et al., 2019, 2020), supplying glutamate to synaptic mitochondria. Interestingly, we found normal fEPSP generation and synaptic plasticity in aGLT-1 KO mice from 6 weeks to 8 months of age, while fEPSP generation and synaptic plasticity were impaired in nGLT-1 KO mice starting in adulthood (17 weeks) (Rimmele et al., 2021). These results suggest that nGLT-1 plays an unexplored role in synaptic function. In the present study, we have found that the effects of soluble oAβ to impair synaptic plasticity appear to be affected by the presence or absence of nGLT-1, but not aGLT-1, as Aβ oligomers fail to inhibit hippocampal LTP in the nGLT-1 KO mice.

It has been reported that Aβ species significantly reduced or altered the expression of GLT-1 in astrocytes (Talantova et al., 2013; Takahashi et al., 2015; Zumkehr et al., 2015), and genetic or pharmacological recovery of GLT-1 alleviated Aβ neuropathology and rescued cognition in AD mouse models (Fan et al., 2018; Takahashi et al., 2015; Zumkehr et al., 2015), suggesting that astrocytic GLT-1 is involved in the pathogenesis of AD (Tong et al., 2017). Previous studies showed that the changes in gene expression in aGLT-1 KO mice were related to inflammation, and these changes were similar to those in the aging human brain and AD (Sharma et al., 2019). The regulation of astrocytic glutamate transporters by pro-inflammatory cytokines has been reported *in vivo* and *in vitro* (Tilleux and Hermans, 2007; Zumkehr et al., 2018). In line with this evidence, many reports have shown that pro-inflammatory cytokines are involved in the pathogenesis of AD (Lau et al., 2021; Thakur et al., 2022). Recent studies also have demonstrated that several pro-inflammatory cytokines regulate GLT-1 (Dumont et al., 2014; Zumkehr et al., 2018). Interestingly, pro-inflammatory cytokines, such as IL-1β and TNF-α, have been reported to inhibit hippocampal LTP (Prieto et al., 2019). Aβ oligomers may trigger inflammatory responses that regulate GLT-1, synaptic function, and cognition. Several GLT-1 activators, such as ceftriaxone and LDN compounds, increase GLT-1 expression and prevent Aβ oligomer-induced neurotoxicity (Foster et al., 2019; Wilkie et al., 2021). In the present study, we did not find that LDN compounds have any protective effect against Aβ-impairment of hippocampal LTP in acute brain slices. This may be due to LDN compounds’ beneficial effect being related to the inflammatory response or upregulating the GLT-1, which may not occur in short-term recording from brain slices.

The pharmacological agents for up-regulating GLT-1 are ceftriaxone and LDN/ OSU-0212320 or LDN/OSU-0215111, reported to increase GLT-1 protein expression *in vitro* in primary astrocytes or following *in vivo* administration (Kong et al., 2014; Foster et al., 2019). Here, we observed ceftriaxone, but not LDN compounds, prevented Aβ oligomers impaired hippocampal LTP. The ceftriaxone effect may not be due to an increase in GLT-1 expression as the treatment is short (**~**4 hours). It has been found that ceftriaxone has many effects on cells, such as activation of NF-κB, increased glutathione, superoxidase dismutase, brain-derived neurotrophic factor level, neurogenesis, and neuronal survival (Tai et al., 2019), and reduces the presynaptic release of glutamate (Wilkie et al., 2021) or activity of group II metabotropic glutamate receptors (Fan et al., 2022). The present result showing that ceftriaxone prevents impairment of LTP by Aβ suggests that Aβ may increase presynaptic glutamate release. It was reported that synthetic Aβ_42_ induces glutamate release (Talantova et al., 2013). Endogenous Aβ has been reported to increase the release probability of excitatory synapses (Abramov et al., 2009; Cummings et al., 2015) and thereby elicit an increase in synaptic glutamate release (Hascup and Hascup, 2016). These data suggest that the pathogenesis of AD, and, specifically, the synaptotoxicity of Aβ may involve a presynaptic mechanism or target within the axon terminal.

EUK-134 is a cell-permeable manganese-containing superoxide and catalase mimic (Rong et al., 1999) that prevents reactive oxygen species (ROS) accumulation (SanMartín et al., 2017). In line with previous reports that nGLT-1 serves as a significant source of glutamate for utilization by synaptic mitochondria (McNair et al., 2019, 2020), restoration of synaptic mitochondrial redox homeostasis restores functional recovery of nGLT-1 KO slices. Our findings suggest that Aβ oligomers’ interruption of synaptic plasticity may involve not only inhibition of glutamate reuptake, but also presynaptic metabolism and mitochondrial ROS production (Torres et al., 2021; Picón-Pagès et al., 2022; Jekabsone et al., 2023). It is also possible that oAβ interacts with many cell membrane receptors/ligands (including GLT-1), leading to activation of various abnormal signaling pathways (such as calcium dysregulation, oxidative stress, and mitochondrial dysfunction), further contributing to impaired synaptic function. Accordingly, the effects of ceftriaxone on preventing impairment of LTP by oAβ may be due to a variety of possible effects, including suppression of excess ROS generation and enhancement of antioxidant defense enzymes (El-Megharbel et al., 2022).

Age represents a significant AD risk factor, with mitochondrial dysfunction being a hallmark of the aging process (Payne and Chinnery, 2015). Slices from the nGLT-1 KO mice showed age-dependent synaptic injury due to glutamate excitotoxicity. Interestingly, soluble oAβ impairment of hippocampal LTP can be rescued in nGLT-1 KO mice. Decreased EAAT2 expression was found in the brains of the elderly (Pereira et al., 2017) and in sporadic AD patients (Jacob et al., 2007; Scott et al., 2011). A decreased EAAT2 activity was associated with synaptic loss in aging (Potier et al., 2010) and neuronal loss in the AD brain (Scott et al., 2011). Although the aGLT-1 KO mice show earlier developing behavioral phenotypes compared with the nGLT-1 KO mice (Sharma et al., 2019), both genotypes develop deficits on Morris Water Maze testing by 17 months of age. We found the preservation of basic synaptic functions and LTP in aGLT-1 KO mice. Impaired synaptic function in adult nGLT-1 KO mice may suggest that nGLT-1 plays a crucial role in hippocampal synaptic plasticity, synaptic metabolism, and synaptic aging. Neuronal GLT-1 may modulate synaptic function by affecting or contributing to presynaptic glutamate release (Borisova and Borysov, 2016; Grewer et al., 2008), synaptic mitochondrial metabolism (McNair et al., 2019), or glutamate uptake. While the loss of aGLT-1 may not be enough to affect stimulus-evoked synaptic responses, spontaneous synaptic release may be altered in aGLT-1 KO mice. Both mitochondrial and synaptic dysfunction are characteristic events of aging (Mattson and Arumugam, 2018). They are reciprocally influenced (Nguyen and Atwood, 1994) and lead to cognitive impairment (Denver and McClean, 2018). The findings from the EUK-134 experiment, which restored synaptic function in nGLT-1 knockout mice and prevented LTP impairment caused by soluble Aβ oligomers, further support the idea that synaptic mitochondrial dysfunction could be a potential predisposing factor for AD. In summary, the present study demonstrates that soluble oAβ impairs hippocampal synaptic plasticity by selectively perturbing one or more functions of GLT-1 localized to axon terminals (**Additional Figure 4**). This effect may be due to altering presynaptic release in the hippocampal CA1 region. These findings provide new insights regarding GLT-1 function and hold potential for the development of new therapeutical targets for the treatment of Alzheimer’s disease.

## Limitations

This study, while contributing valuable insights into the interaction between soluble Aβ oligomers and the glutamate transporter GLT-1, encounters several limitations. First, the model primarily uses conditional GLT-1 knockout mice, limiting the generalizability of findings to natural pathologies in human AD cases where knockout models may not accurately replicate all aspects of AD. Additionally, the experimental conditions for hippocampal slices, such as ACSF preparation and control of oxygen levels, may not fully mimic the *in vivo* environment, potentially affecting the extrapolation of results to live neuronal systems. Another limitation is the short-term nature of slice recordings, as prolonged exposure to Aβ oligomers or GLT-1 modulation could yield additional effects not captured in acute experiments. Lastly, while ceftriaxone and other pharmacological agents were used to modulate GLT-1, potential off-target effects of these agents may complicate the interpretation of results specifically attributed to GLT-1 activity.

## Conclusion

This study demonstrates that soluble Aβ oligomers impair hippocampal LTP by targeting the neuronal isoform of GLT-1 (nGLT-1) rather than astrocytic GLT-1. The findings suggest that the synaptotoxicity of Aβ oligomers may be modulated through mechanisms affecting glutamate reuptake homeostasis at neuronal terminals. Furthermore, the protective effects of ceftriaxone, likely mediated through a presynaptic mechanism, provide a potential avenue for therapeutic intervention aimed at preserving synaptic plasticity in AD. The differential roles of neuronal and astrocytic GLT-1 isoforms underscore the complexity of glutamate regulation in AD pathophysiology, highlighting the importance of targeting specific glutamate transport pathways in the development of future treatments. These insights into nGLT-1’s function advance our understanding of synaptic vulnerabilities in AD and point to promising pharmacological strategies for mitigating Aβ-mediated synaptic dysfunction.

## Additional files:

***Additional Figure 1:*** *Specific glutamate transporter 1 inhibitor WAY213613 fails to prevent soluble amyloid-β oligomers from impairing hippocampal long-term potentiation.*

Additional Figure 1 Specific glutamate transporter 1 inhibitor WAY213613 fails to prevent soluble amyloid-β oligomers from impairing hippocampal long-term potentiation.
Low dose WAY 213613 (20 nM) did not prevent 7PA2 CM (Veh + 7PA2, *n* = 6 *vs*. WAY + 7PA2, *n* = 8) impairment of LTP (one-way analysis of variance with Bonferroni post hoc tests). fEPSP: Field excitatory postsynaptic potentials.

***Additional Figure 2:*** *Hippocampal LTP in nGLT-1 KO mice is resistant to the effects of soluble A*β *oligomers.*

Additional Figure 2 Hippocampal LTP in nGLT-1 KO mice is resistant to the effects of soluble Aβ oligomers.
(A) Slices from young (6-10 weeks old) aGLT-1 KO mice showed normal hippocampal LTP (WT, *n* = 9 *vs*. aKO, *n* = 6, *P* = 0.05), and this LTP was sensitive to 7PA2 CM (purple, *n* = 6) as also in WT slices (red, *n* = 9). (B) Summary data of soluble Aβ oligomers 7PA2 CM and AβS26C dimer (10 nM) impaired hippocampal LTP from young WT and aKO mice (one-way analysis of variance with Bonferroni post hoc tests). (C) Summary data from 6-week-old nGLT-1 KO mouse's effect on different sources of soluble Aβ oligomers (AβS26C dimer (10 nM) and AD brain extract) (one-way analysis of variance with Bonferroni *post hoc* tests). AD: Alzheimer's disease; Aβ: amyloid-β; CM: conditioned medium; fEPSP: field excitatory postsynaptic potentials; GLT-1: glutamate transporter 1; KO: knockout; LTP: long-term potentiation; nGLT-1: neuronal GLT-1; oAβ: soluble Aβ oligomer; TBS: Tris-buffered saline; WT: wild-type.

***Additional Figure 3:*** *Synaptic function recovered brain slices of nGLT-1 KO mice are resistant to the effects of soluble A*β *oligomers.*

Additional Figure 3 Synaptic function recovered brain slices of nGLT-1 KO mice are resistant to the effects of soluble Aβ oligomers.
Recordings of hippocampal long-term potentiation in slices recovered with EUK-134 (30 μM) exposed to AD brain extract yielded results in nGLT-1 KO (red, *n* = 9) but not in WT (black, *n*=6, *P* < 0.05) mice (Student's *t*-test). AD: Alzheimer's disease; Aβ: amyloid-β; GLT-1: glutamate transporter 1; KO: knockout; nGLT-1: neuronal GLT-1; nKO: neuronal GLT-1-KO; oAβ: soluble Aβ oligomer; TBS: Tris-buffered saline; WT: wild-type.

***Additional Figure 4:*** *Soluble amyloid-*β *oligomers impair hippocampal synaptic plasticity by selectively perturbing one or more functions of glutamate transporter 1 localized to axon terminals.*

Additional Figure 4 Soluble amyloid-β oligomers impair hippocampal synaptic plasticity by selectively perturbing one or more functions of glutamate transporter 1 localized to axon terminals.
fEPSP: Field excitatory postsynaptic potentials; HFS: high-frequency stimulation.

***Additional Table 1:*** *Demographic details of cases in this study.*

## Data Availability

*All relevant data are within the paper and its Additional files*.
