# Supplementary figures and images for: Impairment of hippocampal long-term potentiation by soluble amyloid-β oligomers is mediated by glutamate transporter 1 expressed in neurons
Source: Neural Regen Res. 2025 Mar 25;21(7):3171–7. doi: 10.4103/NRR.NRR-D-24-00882 (PMC13378951; doi:10.4103/NRR.NRR-D-24-00882)

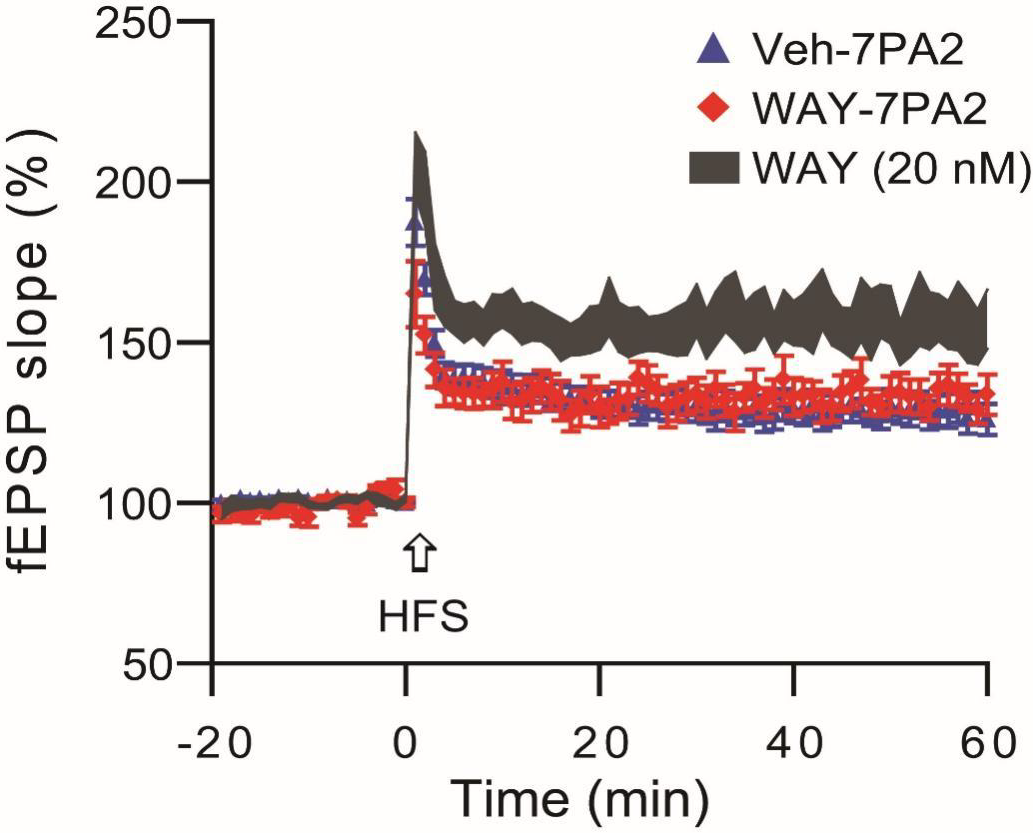

Supplement: Supplementary file 1 [file NRR-21-3171_Suppl1.tif]

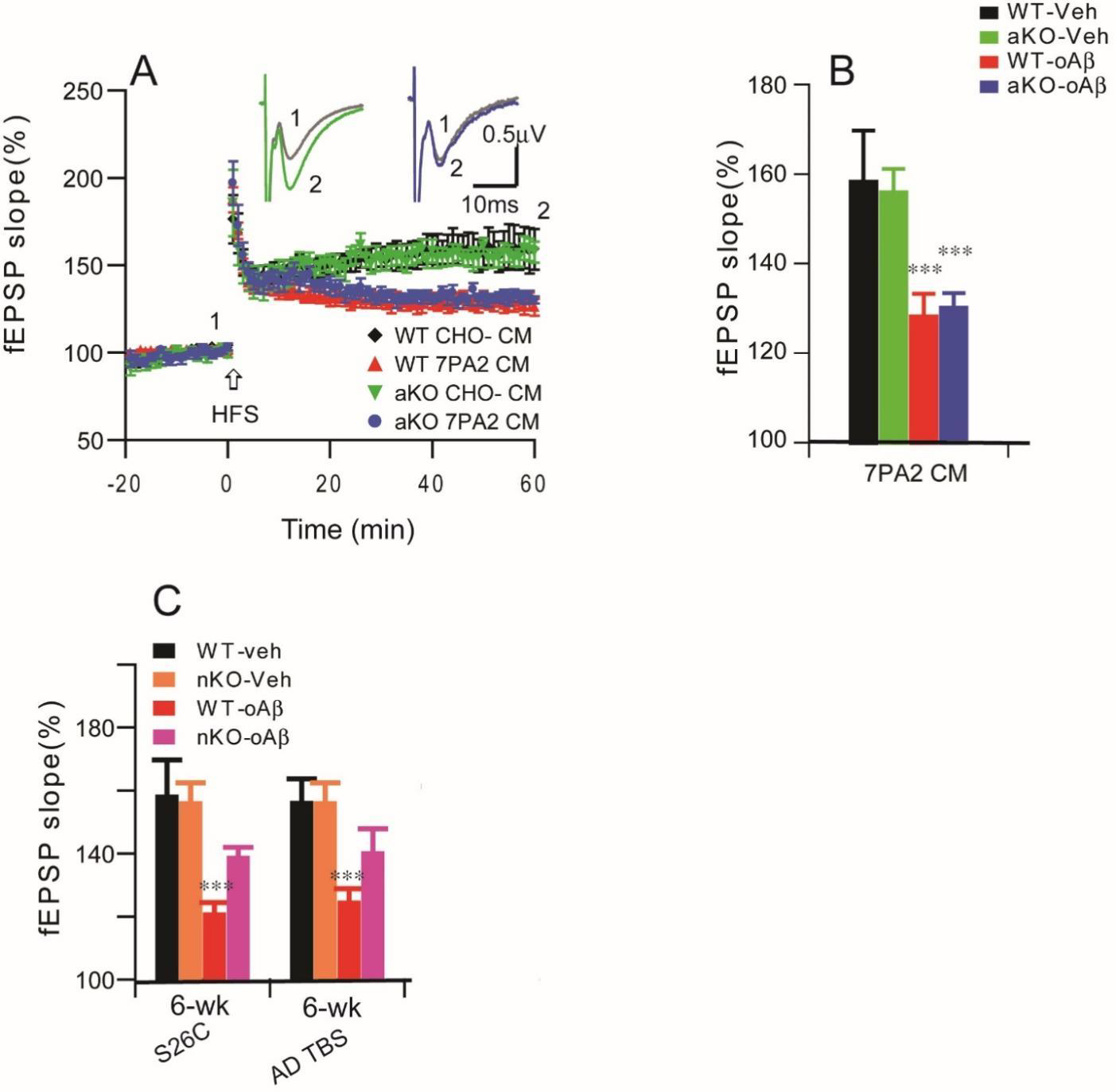

Supplement: Supplementary file 2 [file NRR-21-3171_Suppl2.tif]

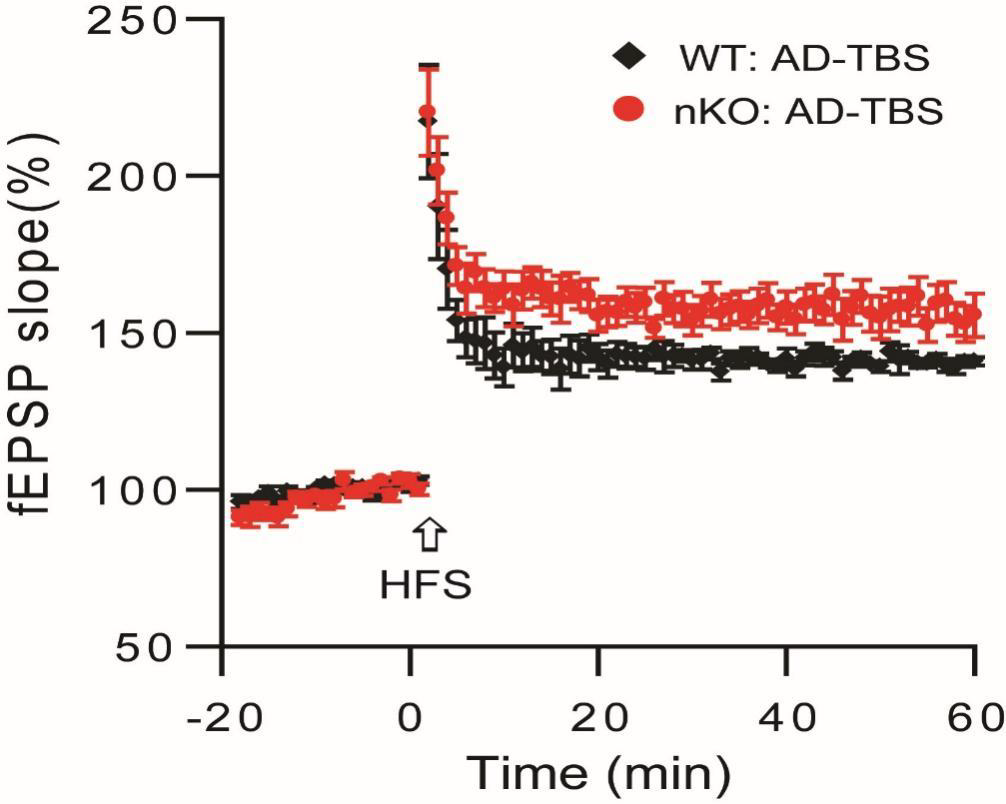

Supplement: Supplementary file 3 [file NRR-21-3171_Suppl3.tif]

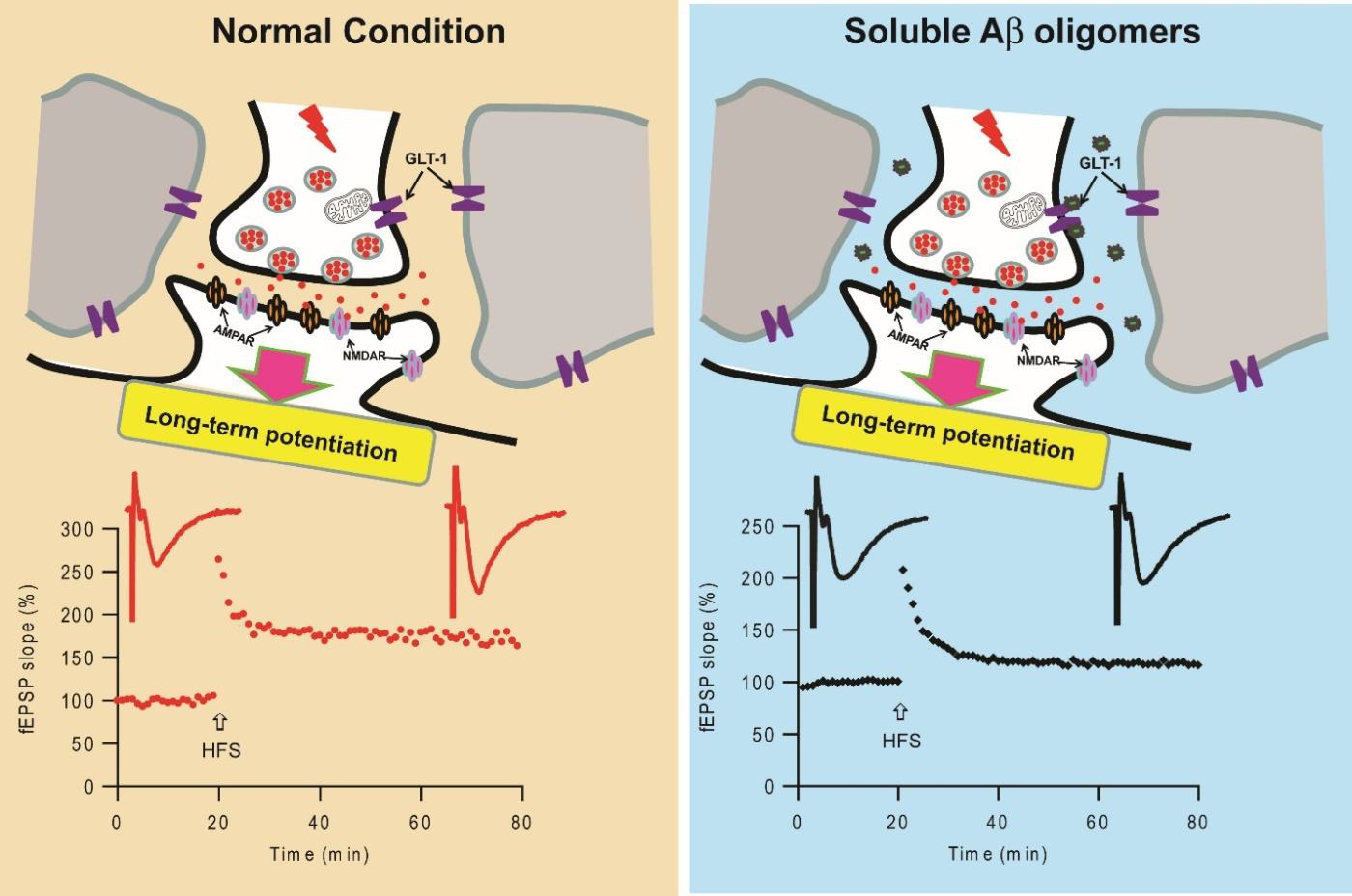

Supplement: Supplementary file 4 [file NRR-21-3171_Suppl4.tif]
